# Cost-effectiveness of ensartinib, crizotinib, ceritinib, alectinib, brigatinib and lorlatinib in patients with anaplastic lymphoma kinase-positive non-small cell lung cancer in China

**DOI:** 10.3389/fpubh.2022.985834

**Published:** 2022-09-21

**Authors:** Xia Luo, Zhen Zhou, Xiaohui Zeng, Liubao Peng, Qiao Liu

**Affiliations:** ^1^Department of Pharmacy, the Second Xiangya Hospital of Central South University, Changsha, China; ^2^Menzies Institute for Medical Research, University of Tasmania, Hobart, TAS, Australia; ^3^Department of Nuclear Medicine/PET Image Center, the Second Xiangya Hospital of Central South University, Changsha, China

**Keywords:** cost-effectiveness, Non-small cell lung cancer, ALK-TKI, ensartinib, domestic anticancer drug, China

## Abstract

**Objective:**

Six anaplastic lymphoma kinase-tyrosine kinase inhibitors (ALK-TKIs), including one domestic (ensartinib) and five imported ALK-TKIs (crizotinib, ceritinib, alectinib, brigatinib, and lorlatinib), have been recommended as first-line treatments for advanced ALK-positive NSCLC in China. This study sought to examine the cost-effectiveness of these six novel therapies in Chinese patients.

**Material and methods:**

We constructed a Markov model to compare the cost-effectiveness of the six ALK-TKIs as a first-line treatment for patients with advanced ALK-positive NSCLC from the perspective of the Chinese healthcare system. Transition probabilities were estimated by synthesizing data from the PROFILE 1,029 trial and a network meta-analysis. Health state utilities and costs were sourced from published literature, publicly available national databases, and local general hospitals. The robustness of model was assessed *via* deterministic sensitivity analyses and probabilistic sensitivity analyses.

**Results:**

Compared with crizotinib, ensartinib achieved additional 0.12 quality-adjusted life-year (QALY) with marginal costs of $3,249, resulting in an incremental cost-effectiveness ratio (ICER) of $27,553/ QALY. When compared with ceritinib and brigatinib, ensartinib achieved additional 0.06 and 0.03 QALYs with substantially reduced costs. When compared with lorlatinib and alectinib, ensartinib was associated with a lower QALY and decreased total costs; the ICERs for lorlatinib and alectinib were $934,101/ QALY and $164,888/ QALY, respectively.

**Conclusion:**

For Chinese patients with advanced ALK-positive NSCLC, ensartinib was a cost-effective option compared with crizotinib, and was a dominant alternative to ceritinib and brigatinib. Although lorlatinib and alectinib were associated with prolonged survival compared with ensartinib, they were less cost-effective than ensartinib due to the overwhelming total costs.

## Introduction

Lung cancer is an aggressive malignancy responsible for nearly one fifth of cancer-related deaths in the world (1). In 2020, lung cancer ranked first among all malignancies in China, with approximately 816,000 new cases and 715,000 related deaths recorded (1). The management of this disease is intractable due to the diversity of histology and cytology (2). Non-small cell lung cancers (NSCLCs) comprise approximately 85% of all lung cancer cases (3). About 5% of NSCLCs are detected to have the rearrangements of anaplastic lymphoma kinase (ALK) gene (4). Multiple generation ALK-tyrosine kinase inhibitors (TKIs) have been developed to target ALK mutations, and most of them have been established as the standard-of-care globally in treating advanced ALK-positive NSCLC globally (5). The latest Chinese society of clinical oncology (CSCO) Guidelines for NSCLC recommend several ALK-TKIs for treating advanced ALK-positive NSCLC in Chinese patients. These include the first-generation crizotinib, the second-generation ceritinib, alectinib, brigatinib and ensartinib, and the third-generation lorlatinib (6).

Crizotinib is an imported ALK-TKI that was firstly approved by the Chinese National Medical Products Administration (NMPA) in 2013 due to its superior clinical efficacy to conventional chemotherapies (7). However, the use of crizotinib is associated with inevitable acquired resistance and a high rate of central nervous system (CNS) metastases (8). Afterwards, many next-generation ALK-TKIs were imported and used as alternatives, to mitigate the safety concerns that arise from crizotinib (9), leading to a rapidly evolving treatment paradigm for advanced ALK-positive NSCLC patients (10–12). Despite the compelling clinical performance of these imported ALK-TKIs, the attendant high costs have substantially reduced their accessibility and affordability among Chinese patients (13).

Recently, the eXalt3 phase 3 clinical trial (Clinical Trials.gov Identifier: NCT02767804) investigated the efficacy of China-developed ALK-TKI ensartinib (second-generation) vs. crizotinib in the first-line setting for advanced ALK-positive NSCLC. They found that ensartinib has superior systemic and intracranial efficacy to crizotinib, as well as an overall favorable safety profile (14). More importantly, the daily cost of ensartinib is much lower than that of imported agents (15). However, whether the China-developed ALK-TKI would provide a similar or better clinical value at a lower cost compared with alternative imported ALK-TKIs remains to be determined. As ensartinib has recently been approved as a first-line treatment for Chinese patients with advanced ALK positive NSCLC, there have been no studies evaluating cost-effectiveness among these six first-line ALK-TKIs.

Therefore, in the current study, we compared the cost-effectiveness of domestic ensartinib vs. all imported ALK-TKIs (crizotinib, ceritinib, alectinib, brigatinib, and lorlatinib) that are recommended by the CSCO Guidelines as a first-line treatment for advanced ALK-positive NSCLC.

## Materials and methods

### Overview

Using TreeAge Pro software (version 2021, https://www.treeage.com/) for mathematical modeling and R software (version 4.1.3, http://www.r-project.org) for survival fitting, we established a cost-effectiveness model to compared first-line ensartinib with crizotinib, ceritinib, alectinib, brigatinib, and lorlatinib.

This study was reported according to the Consolidated Health Economic Evaluation Reporting Standards 2022 (16) and followed the China Guidelines for Pharmacoeconomic Evaluation (2020) (17). Since our study does not involve human subjects, the approval from Chinese ethics review committee was not required.

### Perspective

Our analysis was performed from a perspective of Chinese healthcare system, which refers to weighing the consumption of healthcare resources against the benefits of interventions obtained by patients in the context of the national healthcare system. The national healthcare system in China is a multilevel system, with the basic medical insurance as the pillar and medical aid as the backup, and commercial health insurance, charitable donations, and medical mutual aid activities as Supplementary Services (18).

### Model construction

**Figure 1:**
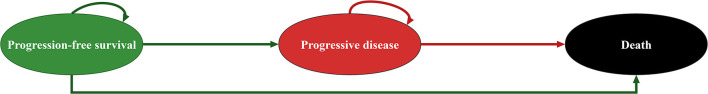
Diagram of markov model.

A Markov model was used to simulate the clinical trajectory of a hypothetical cohort of patients with advanced ALK-positive NSCLC. This model was characterized by 3 main health states: progression-free survival (PFS), progressive disease (PD) and death. Figure 1 illustrates the possible transitions between the three health states. An 8-week cycle period was used to model the real-world intervals between routine follow-ups (6), and a 10-year time horizon was chosen to ensure that all participants in the cohort reached the terminal of death.

All patients entered the model in the PFS health state and were randomized to receive first-line ensartinib (14), crizotinib (8, 19), ceritinib (20), alectinib (10, 21–24), brigatinib (11), and lorlatinib (12) at the dosage detailed in their corresponding clinical trials. During each Markov cycle, patients would stay in the PFS health state, or transfer to corresponding health states when experiencing disease progression or death (Figure 1). Individuals in the PD health state were allowed to receive subsequent anticancer treatments if there were sustainable survival benefits. In addition, patients were supplemented with best supportive care (BSC) when receiving any anticancer therapy and were recommended for palliative care before death (6). Supplementary Appendix 1 provides information regarding the dosage used for each first-line treatment.

The primary model outputs were quality-adjusted life-year (QALY) and cost in 2021 US dollars (1 United States dollars was equivalent to 6.4515 Chinese yuan) (25). We used these outputs to calculate incremental cost-effectiveness ratios (ICERs) between domestic ALK-TKI ensartinib and the imported ALK-TKIs. A treatment strategy was deemed as a cost-effective option if it produced an ICER lower than the willingness-to-pay (WTP) threshold of $37,654 per QALY (3 times of China's per capita GDP in 2021) (17, 25); and a strategy was deemed as the dominant strategy if it was associated with a higher QALY at lower costs. Both costs and QALYs were discounted at 5% annually (17).

### Survival and health state utilities

Indirect comparisons of the six ALK-TKIs were conducted using the survival data of crizotinib from the PROFILE 1,029 clinical trial, because crizotinib is a commonly used comparator in the clinical trials of next-generation ALK-TKIs (10–12, 14). As described in our previous cost-effectiveness studies (26, 27), to calculate the transition probabilities for first-line crizotinib, we first extracted the overall survival (OS) and PFS data from the published Kaplan-Meier curves to reconstruct the patient-level data, which were then fitted and extrapolated with Weibull survival distribution according to the results of goodness-of-fit measures (Supplementary Appendix 2). The final Weibull scale (λ) and shape (γ) parameters were used to estimate the survival probability S(t) at a given time cycle t:.


$$\begin{array}{l} S\left(t\right)=\exp\left(-\lambda t^{\gamma }\right) \end{array}$$

Given the absence of head-to-head clinical trials that directly compared these six ALK-TIKs, we used the HRs of OS and PFS for ensartinib, ceritinib, alectinib, brigatinib, and lorlatinib vs. crizotinib extracted from a published network meta-analysis (NMA) (28) to estimate the transition probabilities for all ALK-TIKs except for crizotinib. The transition probabilities of the other five ALK-TIKs were obtained using the adjusted Weibull scale λ_*other*−*ALK*−*TKIs*_ = λ_*crizotinib*_ × *HR* and shape γ_*other*−*ALK*−*TKIs*_ = γ_*crizotinib*_ parameters.

Utility assigned to each health state was obtained from a previous study that applied a Chinese-specific value algorithm to the EuroQol five-dimension (EQ-5D) questionnaire data (29). In addition, the utility decrements that arise from grade III/IV AEs were also estimated in the model (30). Briefly, the disutility associated with crizotinib was included as a frequency-weighted sum based on the reported grade III/IV AEs data in the PROFILE 1,029 trial; disutilities associated with ensartinib, ceritinib, alectinib, brigatinib, and lorlatinib were calculated using the HRs for AEs derived from the NMA mentioned above (28). The algorithm used to estimate utility decrements is provided in Supplementary Appendix 3, and all survival and health state utilities parameters are summarized in Table 1.

**Table 1:** Model inputs.

**Parameters**	**baseline value**	**Ranges**	**Distribution**	**Source**
**Survival**	
OS for crizotinib	Weibull: λ = 0.02092; γ = 1.25579	Fixed in DSA	Fixed in PSA	Estimated^a^
PFS for crizotinib	Weibull: λ = 0.08242; γ = 1.21862	Fixed in DSA	Fixed in PSA	Estimated^a^
HR_OS_ for ensartinib vs. crizotinib	0.88	0.45–1.73	Normal	Ma HC, et al.
HR_PFS_ for ensartinib vs. crizotinib	0.48	0.20–1.21	Normal	Ma HC, et al.
HR_OS_ for ceritinib vs. crizotinib	0.90	0.44–1.82	Normal	Ma HC, et al.
HR_PFS_ for ceritinib vs. crizotinib	1.28	0.44–3.84	Normal	Ma HC, et al.
HR_OS_ for alectinib vs. crizotinib	0.67	0.38–1.18	Normal	Ma HC, et al.
HR_PFS_ for alectinib vs. crizotinib	0.41	0.21–0.77	Normal	Ma HC, et al.
HR_OS_ for brigatinib vs. crizotinib	0.92	0.49–1.74	Normal	Ma HC, et al.
HR_PFS_ for brigatinib vs. crizotinib	0.49	0.20–1.23	Normal	Ma HC, et al.
HR_OS_ for lorlatinib vs. crizotinib	0.72	0.36–1.43	Normal	Ma HC, et al.
HR_PFS_ for lorlatinib vs. crizotinib	0.28	0.11–0.69	Normal	Ma HC, et al.
**Costs**	
Ensartinib cost per day	59.29	17.79–71.15	Gamma	Local charge
Crizotinib cost per day	70.93	21.28–85.12	Gamma	Local charge
Ceritinib cost per day	105.40	31.62–126.48	Gamma	Local charge
Alectinib cost per day	84.32	25.30–101.19	Gamma	Local charge
Brigatinib cost per day	305.30	91.59–366.36	Gamma	Local charge
Lorlatinib cost per day	209.25	62.78–251.10	Gamma	Local charge
Routine follow-up cost per cycle	383.63	306.91–460.36	Gamma	Local charge
Subsequent anticancer therapy cost per cycle	2,277.47	1,821.98–2,732.96	Gamma	Liu Q, et al.
BSC cost per cycle	900.00	720.00–1,080.00	Gamma	Liu Q, et al.
Palliative care per cycle	7,007.47	5,605.98–8,408.96	Gamma	Liu Q, et al.
AEs cost for ensartinib	798.62	638.90–958.35	Gamma	Estimated^b^
AEs cost for crizotinib	566.40	453.12–679.68	Gamma	Estimated^b^
AEs cost for ceritinib	2,639.42	2,111.53–3.167.30	Gamma	Estimated^b^
AEs cost for alectinib	345.50	276.40–414.60	Gamma	Estimated^b^
AEs cost for brigatinib	724.99	579.99–869.99	Gamma	Estimated^b^
AEs cost for lorlatinib	1,200.77	960.61–1,440.92	Gamma	Estimated^b^
**Utilities**	
PFS health state	0.856	0.718–0.994	Beta	Shen Y, et al.
PD health state	0.768	0.595–0.941	Beta	Shen Y, et al.
Disutility for ensartinib	0.046	0.037–0.055	Beta	Estimated^b^
Disutility for crizotinib	0.033	0.026–0.039	Beta	Estimated^b^
Disutility for ceritinib	0.152	0.122–0.182	Beta	Estimated^b^
Disutility for alectinib	0.020	0.016–0.024	Beta	Estimated^b^
Disutility for brigatinib	0.042	0.033–0.050	Beta	Estimated^b^
Disutility for lorlatinib	0.069	0.055–0.083	Beta	Estimated^b^
**Others**	
Discount rate (%)	5	0–8	Fixed in PSA	Guidelines

DSA, deterministic sensitivity analyses; PSA, probabilistic sensitivity analyses; OS, overall survival; PFS, progression-free survival; HRs, hazard ratios;BSC, best supportive care; AEs, adverse events; PD, progressive disease.

a 6The Weibull distribution parameters, scale (λ) and shape (γ) were estimated based on survival data reported in the PROFILE 1029 trial.

b Estimated in the Supplementary Appendix 3.

### Cost

Drugs acquisition, AEs management, routine follow-ups, subsequent anticancer therapies, BSC and palliative care costs were included in the model. The daily costs of these six items were converted by their prices according to daily dose. The prices of ensartinib, crizotinib, ceritinib, alectinib and lorlatinib were obtained from the latest available bid-winning price in the public national databases (31); the price of brigatinib came from the Hong Kong Hospital Authority because brigatinib has just been approved by the NMPA on March 24, 2022, and its price in Chinese mainland has not yet been released. Similar to disutility, we calculated the AEs management cost for first-line crizotinib by multiplying the frequency of III/IV AEs and the costs corresponding to each AEs investigated from local general hospitals; the calculation of AEs costs for the other five ALK-TIKs is described in Supplementary Appendix 3.

Costs of routine follow-up costs (comprising of history, physical examination and radiological imaging cost recommended by the CSCO Guidelines) were also estimated using data from local general hospitals. In addition, subsequent anticancer treatment, BSC and palliative care costs were extracted from previous literature (26).

### Sensitivity analysis

Two sensitivity analyses were carried out to test the robustness of our conclusion. Deterministic sensitivity analyses (DSA) were performed by varying individual parameters to identify sensitive factors. HRs and health state utilities varied across the published 95% confidence intervals (CIs), ALK-TKIs costs varied within specific ranges based on the Chinese national condition, other costs and disutility varied between the plus and minus 20% of the baseline values, and discounted rate varied from 0 to 8%. Probabilistic sensitivity analyses (PSA) were performed by varying multiple parameters simultaneously. Each parameter was matched with an appropriate statistical distribution, and 10,000 Monte Carlo simulations were used to generate 10,000 model outputs. Table 1 outlines the varying ranges and distributions matched with each parameter.

## Results

### Incremental cost-effectiveness ratios

For Chinese patients with advanced ALK-positive NSCLC, first-line ensartinib achieved additional 0.12, 0.06 and 0.03 QALYs in comparison with crizotinib, ceritinib, and brigatinib, respectively. The incurred total cost associated with ensartinib was marginally greater than crizotinib ($81,283 vs. $78,033), but slightly lower than ceritinib ($81,283 vs. $95,049) and substantially lower than brigatinib ($81,283 vs. $252,984) (Table 2). These results suggested that ensartinib was a cost-effective option compared with crizotinib (ICER = $27,553/ QALY) and a dominant alternative to ceritinib and brigatinib, when the WTP threshold was set at $37,654 per QALY in this analysis.

**Table 2:** Summary of simulation results.

**Outputs**	**Cost (US$)**	**QALYs**	**Incremental**			**ICER $/QALY**
			**Analyses**	**Cost, (US$)**	**QALYs**	
Ensartinib	81,283	1.03	
Crizotinib	78,033	0.91	Ensartinib vs. Crizotinib	3,249	0.12	27,553 (cost-effective)
Ceritinib	95,049	0.97	Ensartinib vs. Ceritinib	−13,766	0.06	Dominance
Brigatinib	252,984	0.99	Ensartinib vs. Brigatinib	−171,701	0.03	Dominance
Lorlatinib	259,939	1.22	Lorlatinib vs. Ensartinib	178,656	0.19	934,101 (not cost-effective)
Alectinib	118,900	1.25	Alectinib vs. Ensartinib	37,617	0.23	164,888 (not cost-effective)

QALY, quality-adjusted life-year; ICER, incremental cost-effectiveness ratio. The symbol “$” stands for US dollars.

Moreover, ensartinib was associated with inferior effectiveness and lower total costs compared with lorlatinib and alectinib. The ICER between lorlatinib and ensartinib was $934,101/ QALY and between alectinib and ensartinib was $164,888/ QALY. These results indicated that lorlatinib and alectinib were not cost-effective compared with ensartinib.

### Sensitivity analysis

**Figure 2:**
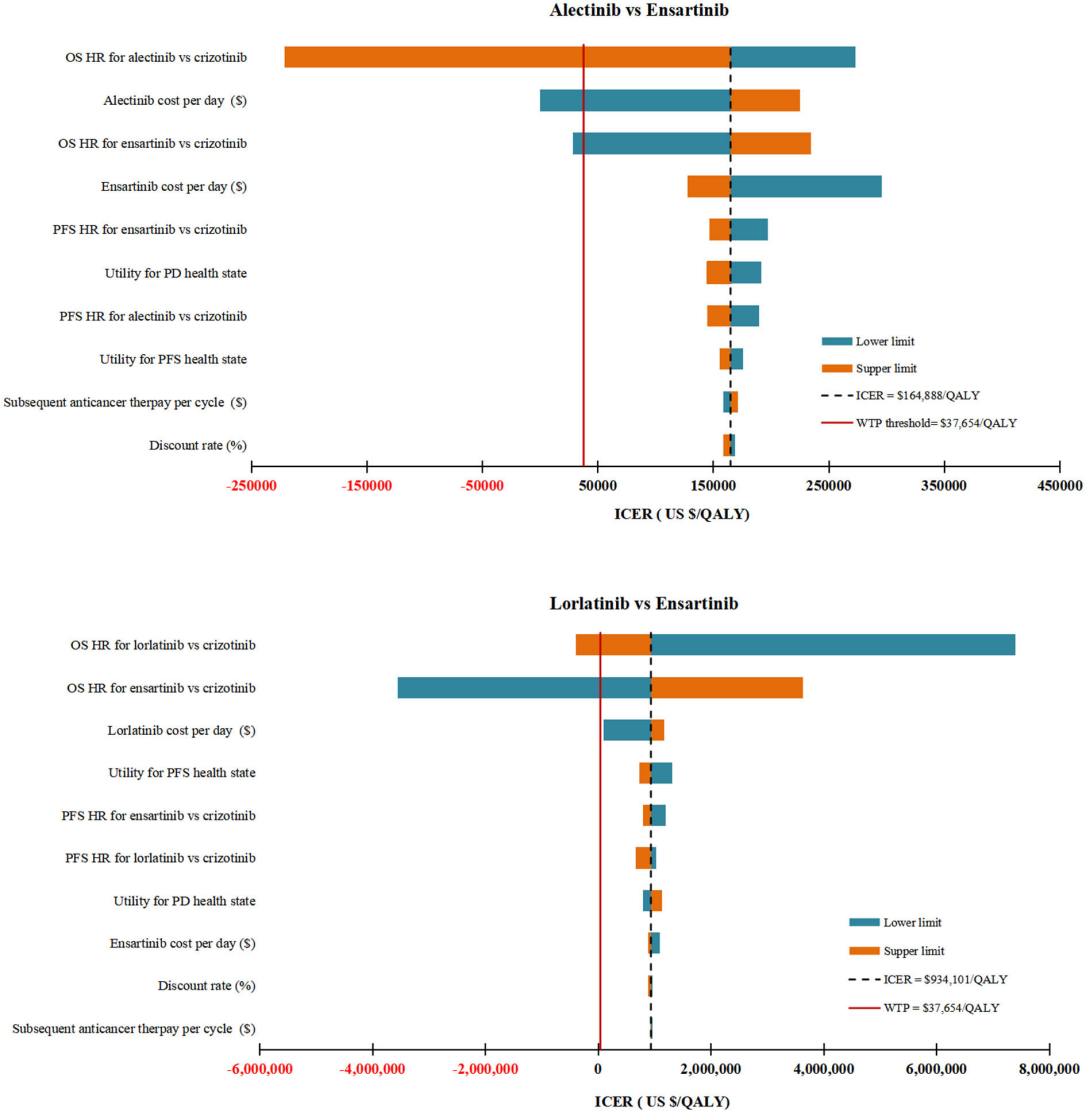
Deterministic sensitivity analysis results. The top 10 parameters by magnitude of effect on the ICER were presented. ICER, incremental cost-effectiveness ratios; QALY, quality-adjusted life-years; WTP, willingness-to-pay; OS, overall survival; PFS, progression-free survival; PD, progressive disease; HR, hazard ratios; BSC, best supportive care.

In DSA for alectinib vs. ensartinib, the model was particularly sensitive to the HR_OS_ for alectinib vs. crizotinib, the daily cost of alectinib and the HR_OS_ for ensartinib vs. crizotinib. To be more specific, decreasing the HR_OS_ for alectinib vs. crizotinib from 0.67 to 0.42, decreasing the daily cost of alectinib from $84.33 to $48.74 and increasing the HR_OS_ for ensartinib vs crizotinib from 0.88 to 1.68, would allow the ICERs under the WTP threshold of $37,654 per QALY. Other model parameters had marginal influence on the model as illustrated by Figure 2.

In DSA for lorlatinib vs. ensartinib, the two parameters with the ability to reverse our model results were the HR_OS_ for lorlatinib vs. crizotinib and the HR_OS_ for ensartinib vs. crizotinib. All other parameters varied but did not yield substantial changes in the ICER (Figure 2). A daily cost of lorlatinib less than $67.28 would make the ICER between lorlatinib and ensartinib ($843,496/QALY) close to the WTP threshold.

The results of PSA showed that both alectinib and lorlatinib were not cost-effective in almost all 10,000 iterations when compared with ensartinib.

## Discussion

In China, healthcare expenditures associated with cancer treatments amounted to a whopping US $30 billion in 2015 and is expected to continue to rise with the ever-increasing cancer cases (32). The substantial financial burdens have been imposed on both patients at an individual level and the national healthcare system at a population level. Since lung cancers are the most common and fatal malignant tumors in China (33), Chinese government, academia and the general public have attached increasing importance to value-based oncology. Although a variety of imported ALK-TKIs have been recommended for Chinese patients with advanced ALK-positive NSCLC, their prohibitive costs discourage Chinese patients from using them despite in an urgent need. This has promoted the emergence of the cheap domestic ALK-TKIs that are affordable and accessible to much more patients. However, as there is no authoritative cost-effectiveness evidence to inform whether domestic ALK-TKI is preferable to be used compared with imported ALK-TKIs, it is challenging for physicians to make appropriate evidence-based clinical decisions.

In this cost-effectiveness analysis, we comprehensively compared the effectiveness and cost of domestic ALK-TKI ensartinib with five imported ALK-TKIs in the first-line treatment of Chinese patients with advanced ALK-positive NSCLC. Our data suggested that ensartinib was a cost-effective option compared with crizotinib and a dominant alternative to ceritinib and brigatinib. Moreover, although lorlatinib and alectinib were associated with extended survival compared with ensartinib, their overwhelming costs made them to be less cost-effective than ensatinib. To our best knowledge, this is the first cost-effectiveness analysis studying the first domestic ALK-TKI ensartinib approved as a frontline therapy for Chinese patients with advanced ALK-positive NSCLC, and it is also the first one to investigate all the six ALK-TKIs (ensartinib, crizotinib, ceritinib, alectinib, brigatinib, and lorlatinib) recommended by the CSCO Guidelines for treating advanced ALK-positive NSCLC in Chinese patients.

Our results demonstrated favorable effectiveness associated with alectinib and lorlatinib, which were consistent with previous clinical trials (10, 21–24). The median PFS of alectinib in two large-scale, phase 3, crizotinib-controlled randomized clinical trials exceeded 30 months, while the median PFS of ceritinib, brigatinib, and ensartinib were 16.6 months, 24.0 months, and 25.8 months, respectively (11, 14, 20). As for lorlatinib, despite the immature PFS data, the lowest reported HR of PFS for the third-generation ALK-TKIs vs. crizotinib to data seems to indicate its superior PFS (12). In term of OS, although the median OS for alectinib and lorlatinib were not reached, the clinically significant differences in OS rate (12, 21), the better intracranial lesion control (12, 21), and the potential correlation between PFS benefits and OS benefits (34, 35), may translate into their better survival benefits. Therefore, we could expect that for alectinib and lorlatinib, there are trends toward improved OS. This assumption is also supported by the relatively low HR generated by the NMAs (28, 36, 37).

Sensitivity analyses revealed that HR OS plays a decisive role in determining whether the studied ALK-TKI is cost-effective or not. Due to the lack of clinical trial that directly compares these six ALK-TKIs, HRs from a systematic NMA were used for transition probabilities estimation in this economic evaluation. This NMA comparatively summarized the effectiveness and safety of several first-line ALK-TKIs by synthesizing the clinical data from all randomized controlled trials completed to date (28). Apart from the HRs, the price of ALK-TKIs is another important parameter that considerably influenced our results. In our sensitivity analysis, we set the lowest limit of ALK-TKIs to 30% of the original price based on China's national conditions. To meet the growing demand for cancer treatment, reducing the price of anticancer drugs has always been the Chinese government's top priorities (38). In recent years, the Chinese government has promulgated many effective medical reform policies. The most representative and influential ones included bolstering research and development of domestic anticancer drugs (39), negotiating the price of anticancer drugs with suppliers (40), and reimbursing anticancer drugs through national medical insurance (41). Driven by national policies, numerous domestic anticancer drugs such as ensartinib have come into the market in China, and many anticancer drugs have achieved price reduction of more than 50% (42).

Since the price of brigatinib is not available in Chinese mainland market, we used the price from the Hong Kong Hospital Authority to inform the cost estimation. Results from this cost-effectiveness model suggested that brigatinib was dominated by ensartinib due to a lower QALY and a greater total costs. Moreover, given the price advantage of domestic drugs and the government support, the listing price of brigatinib in Chinese mainland would unlikely be lower than that of ensartinib. Therefore, we could conservatively conclude that brigatinib does not represent as a cost-effective strategy compared with ensartinib. As for loratinib approved on April 27th, considering its superior efficacy in prolonging survival over ensartinib, we performed separate sensitivity analyses to explore the sensitive factors related to its cost-effectiveness. We found that compared with ensartinib, loratinib was not a preferred strategy regardless of its daily cost.

This study has some limitations. First, although the indirect cost-effectiveness comparisons of these six ALK-TKIs were based on a well-designed comprehensive NMA analysis, further validation of our findings is necessary when head-to-head clinical trial data are available. Second, owing to the dearth of quality-of-life data specifically applicable to Chinese patients with advanced ALK-positive NSCLC, we used the health state utilities measured for advanced NSCLC patients from a Chinese-based study (28); however, our results were hardly influenced by the uncertainty in utilities. Third, the resources used to inform cost estimations in this study were not real-time statistics, such as subsequent anticancer treatment, BSC and palliative care costs; our sensitivity analysis indicated that our model was not sensitive to these particular inputs. Fourth, given the disparities in model inputs and study perspectives, the applicability and generalizability of our findings to other countries may be limited; however, given that China contributes approximately 40% of the newly diagnosed NSCLC cases worldwide (1, 33), the cost-effectiveness evidence yielded from this unique study will be useful to help alleviate both national and global cancer burdens. Finally, due to the in-trial crossover in the ALTA-1L study comparing the efficacy and safety of brigatinib with crizotinib in patients with advanced ALK-positive NSCLC (11), the OS benefit associated with brigatinib may be underestimated. However, this potential underestimation on the efficacy of brigatinib is unlikely to change our conclusion due to the substantially higher total cost of brigatinib than ensartinib.

In conclusion, in this economic evaluation comparing the domestic ALK-TKI ensartinib with five imported ALK-TKIs for advanced ALK-positive NSCLC in Chinese patients, ensartinib was a cost-effective option compared with crizotinib, but a dominant alternative to ceritinib and brigatinib. Moreover, despite lorlatinib and alectinib showing superior efficacy in prolonging survival over ensartinib, their overwhelming costs made them less cost-effective than ensartinib.

## Data availability statement

The original contributions presented in the study are included in the article/Supplementary material, further inquiries can be directed to the corresponding author.

## Author contributions

QL: had full access to all of the data in the study and took responsibility for the integrity of the data and the accuracy of the data analysis. XL, XZ, and LP: concept and design. XL and ZZ: drafting of the manuscript. QL: statistical analysis. XZ and XL: obtained funding. All authors: acquisition, analysis, or interpretation of data and critical revision of the manuscript for important intellectual content. All authors contributed to the article and approved the submitted version.

## Funding

This work was supported by the Hunan Provincial Natural Science Foundation [grant number 2021JJ40817]; Hunan Provincial Natural Science Foundation [grant number 2021JJ80080].

## Conflict of interest

The authors declare that the research was conducted in the absence of any commercial or financial relationships that could be construed as a potential conflict of interest.

## Publisher's note

All claims expressed in this article are solely those of the authors and do not necessarily represent those of their affiliated organizations, or those of the publisher, the editors and the reviewers. Any product that may be evaluated in this article, or claim that may be made by its manufacturer, is not guaranteed or endorsed by the publisher.
