# Supplementary material for: Cost-effectiveness of ensartinib, crizotinib, ceritinib, alectinib, brigatinib and lorlatinib in patients with anaplastic lymphoma kinase-positive non-small cell lung cancer in China
Source: Front Public Health. 2022 Sep 21;10:985834. doi: 10.3389/fpubh.2022.985834 (PMC9533130; doi:10.3389/fpubh.2022.985834)
Supplement: Supplementary file 1 [file Data_Sheet_1.docx]

**Supplementary Appendix 1 First-line treatments used in the model.**

The dosage and schedule of each anaplastic lymphoma kinase (ALK)-tyrosine kinase inhibitors (TKIs) were source from representative phase III clinical trials.

**Table S1 First-line treatments used in the model**

| **Regimens** | **Dosage and schedule** |
| --- | --- |
| Ensartinib | 225mg once daily |
| Crizotinib | 250mg twice daily |
| Ceritinib | 750 mg once daily |
| Alectinib | 600mg twice daily |
| Brigatinib | 90mg once daily for the first 7 days, and 180 mg daily thereafter |
| Lorlatinib | 100mg once daily |

**Supplementary Appendix 2** **The selection of survival distributions for first-line crizotinib**

We first extracted the survival data from the published Kaplan-Meier curves to reconstruct the patient-level data, which were then fitted and extrapolated with five commonly used parametric survival distributions, including exponential, Weibull, log-normal, log-logistic and gompertz distributions. Next, based on the results of goodness-of-fit measures [Akaike’s information criterion (AIC) and Bayesian information criterion (BIC)] and visual fit, we selected the optimal survival model for first-line crizotinib.

**OS Fit**

As for the overall survival (OS) Kaplan-Meier curves of first-line crizotinib, the visual fits of the five mentioned-above parametric survival distributions was illustrated in **Figure S1**, and the AIC and BIC statistics were detailed in **Table S2**. Although the visual fits showed that these five distributions provided similar fits for published OS data, exponential, log-normal,log-logistic distributions produced higher extended tails, which implied an overestimation of OS in the long-term. Meanwhile, based on the AIC and BIC statistics, the Weibull distribution may be appropriate as it provided the lowest values.

**
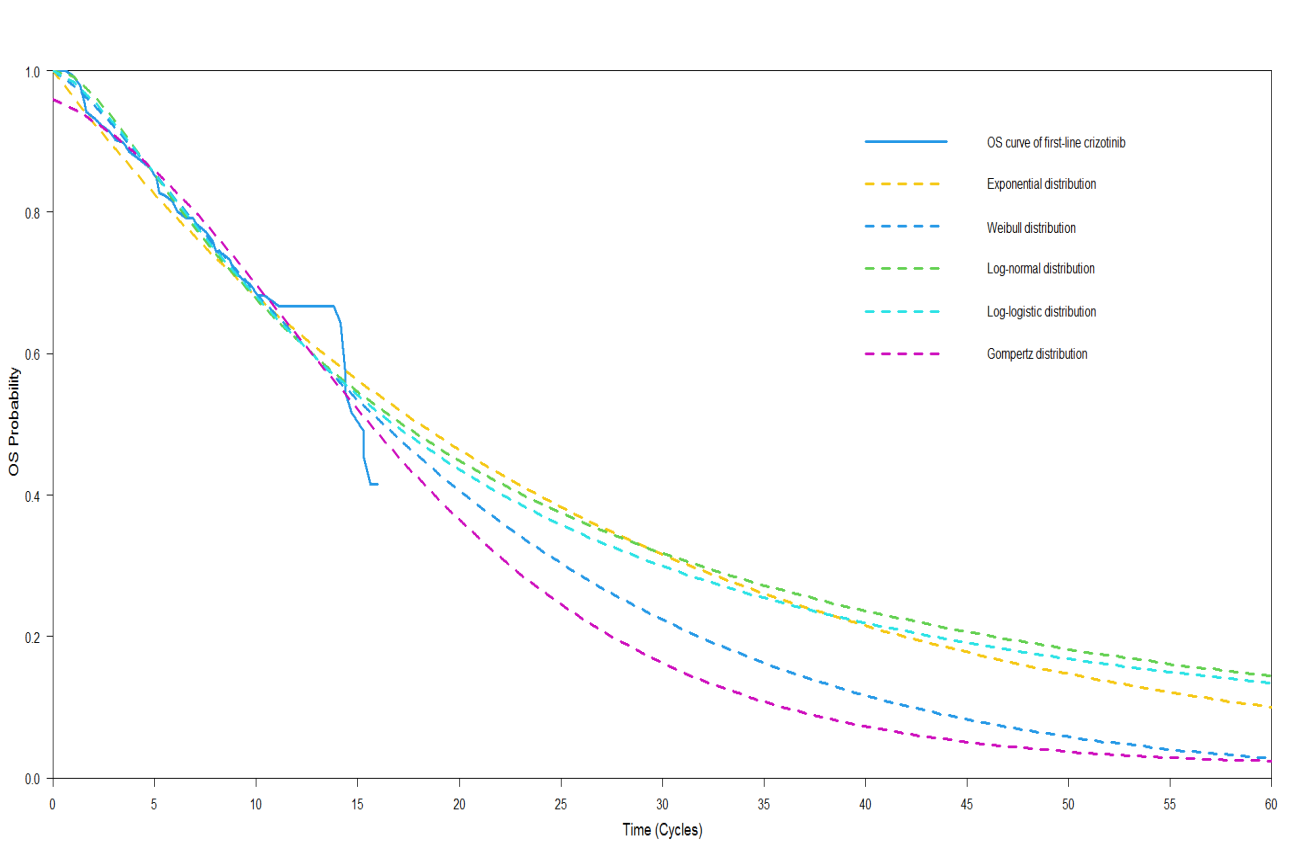
**

**Figure S1 Parametric survival distributions fitting for OS data of first-line crizotinib**

**Table S2 AIC and BIC statistics for OS data of first-line crizotinib.**

| Parametric survival distribution | **OS data** | |
| --- | --- | --- |
|  | **AIC** | **BIC** |
| Exponential | -155 | -155 |
| Weibull | **-165** | **-165** |
| Log-normal | -156 | -156 |
| Log-logistic | -160 | -160 |
| Gompertz | -159 | -159 |

*OS, overall survival; AIC, Akaike information criterion; BIC, Bayesian information criterion.*

**PFS Fit**

As for the progression-free survival (PFS) Kaplan-Meier curves of first-line crizotinib, the visual fits of the five mentioned-above parametric survival distributions was illustrated in **Figure S2**, and the AIC and BIC statistics were detailed in **Table S3**. The visual fits indicated that these five distributions produced similar fits for published PFS data. We chosen the Weibull survival distribution for the PFS data of first-line crizotinib because its lowest BIC and AIC.


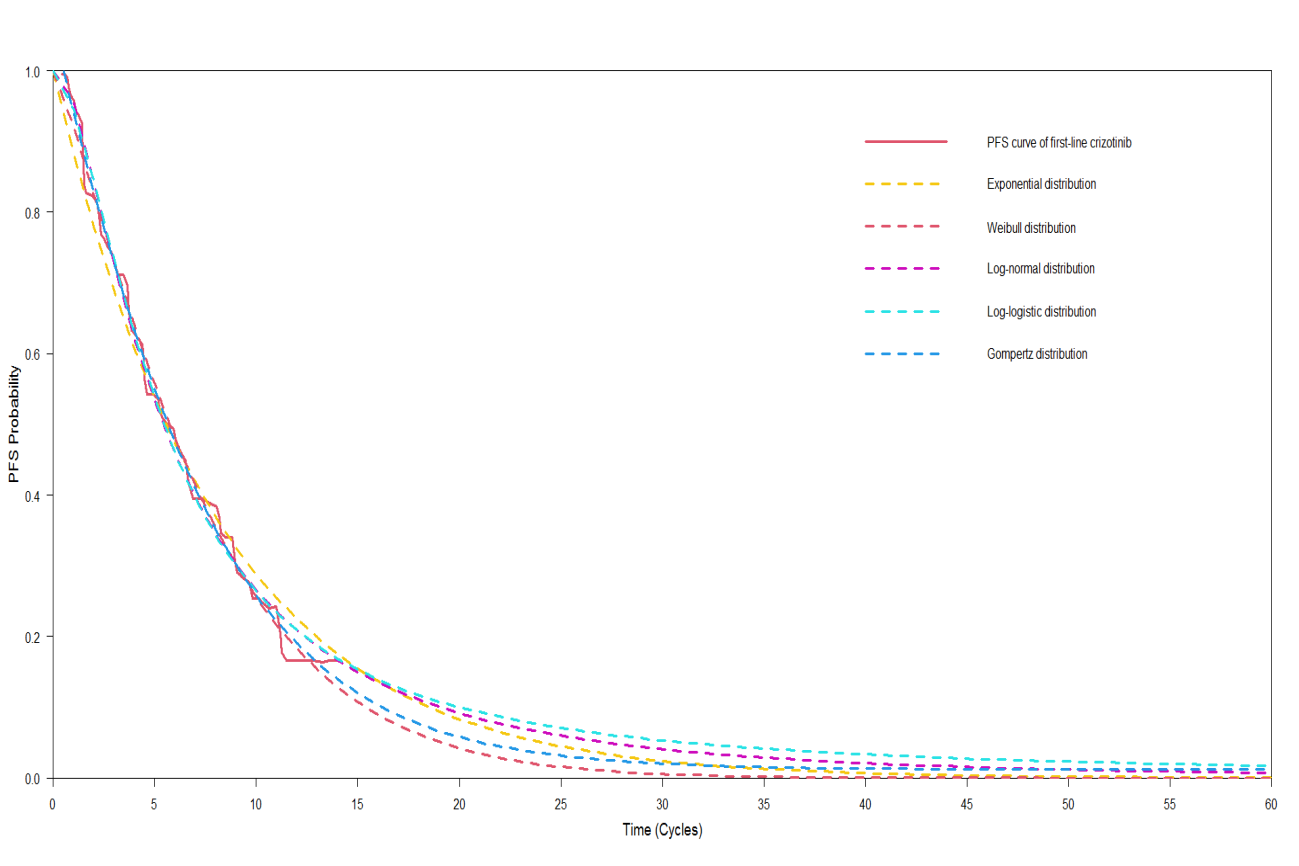


**Figure S2 Parametric survival distributions fitting for PFS data of first-line crizotinib**

**Table S3 AIC and BIC statistics for PFS data of first-line crizotinib.**

| Parametric survival distribution | **PFS data** | |
| --- | --- | --- |
|  | **AIC** | **BIC** |
| Exponential | -250 | -246 |
| Weibull | **-337** | **-330** |
| Log-normal | -325 | -318 |
| Log-logistic | -326 | -319 |
| Gompertz | -295 | -286 |

**Supplementary Appendix 3 Proportion, costs and disutility of grade III/IV AEs considered in the model**

The incidence of grade III/IV adverse reactions (AEs) associated with first-line crizotinib was obtained from the PROFILE 1029 clinical trial, and the hazard ratios of grade III/IV AEs of other five next-generation ALK-TIKs relative to crizotinib were derived from the published network meta-analysis conducted by Ma HC et al.

**Table S4 Proportion, costs and disutility of grade III/IV AEs considered in the model.**

| AEs | First-line crizotinib (%) | HRs | | | | | Cost per event($)^a^ | Disutility |
| --- | --- | --- | --- | --- | --- | --- | --- | --- |
|  |  | ensartinib vs crizotinib | ceritinib vs crizotinib | alectinib vs crizotinib | brigatinib vs crizotinib | lorlatinib vs crizotinib |  |  |
| Neutropenia | 16.3% | 1.41  (0.22,9.27) | 4.66 (0.46, 54.10) | 0.61  (0.16,2.28) | 1.28 (0.18,8.53) | 2.12 (0.32,13.88) | 1094.28 | 0.20 |
| Elevated transaminases | 11.5% |  |  |  |  |  | 292.59 | /^b^ |
| Anemia | 2.9% |  |  |  |  |  | 2150.12 | /^b^ |
| Leukopenia | 2.9% |  |  |  |  |  | 1267.73 | /^b^ |
| Pneumonia | 2.9% |  |  |  |  |  | 1229.23 | /^b^ |
| Dyspnea | 1.9% |  |  |  |  |  | 8949.61 | /^b^ |
| Pleural effusion | 1.0% |  |  |  |  |  | 478.36 | /^b^ |
| Thrombocytopenia | 1.9% |  |  |  |  |  | 1415.63 | /^b^ |
| Hyponatremia | 1.0% |  |  |  |  |  | 316.83 | /^b^ |
| Hypokalemia | 1.9% |  |  |  |  |  | 775.05 | /^b^ |
| Estimated AEs Costs and disutilities^c^ |  |  |  |  |  |  |  |  |
| AEs cost for first-line crizotinib, $ |  |  |  |  |  |  | 566.40 |  |
| AEs cost for first-line ensartinib, $ |  |  |  |  |  |  | 798.62 |  |
| AEs cost for first-line ceritinib, $ |  |  |  |  |  |  | 2639.42 |  |
| AEs cost for first-line alectinib, $ |  |  |  |  |  |  | 345.50 |  |
| AEs cost for first-line brigatinib, $ |  |  |  |  |  |  | 724.99 |  |
| AEs cost for first-line lorlatinib, $ |  |  |  |  |  |  | 1200.77 |  |
| AEs disutility for first-line crizotinib |  |  |  |  |  |  |  | 0.033 |
| AEs disutility for first-line ensartinib |  |  |  |  |  |  |  | 0.046 |
| AEs disutility for first-line ceritinib |  |  |  |  |  |  |  | 0.932 |
| AEs disutility for first-line alectinib |  |  |  |  |  |  |  | 0.020 |
| AEs disutility for first-line brigatinib |  |  |  |  |  |  |  | 0.042 |
| AEs disutility for first-line lorlatinib |  |  |  |  |  |  |  | 0.069 |

*AEs, adverse events; HR, hazard ratios.*

*^a^These AEs management costs used to inform model were investigated from local comprehensive hospitals.*

*^b^The disutility regarding these AEs were not reported.*

*^c^The AEs Costs and disutilities associated with ensartinib, ceritinib, alectinib, brigatinib and lorlatinib were calculated by multiplying the AEs HRs of these five ALK-TIKs compared with crizotinib povided in the network network meta-analysis by the corresponding AEs Costs and disutility associated with crizotinib.*
